# Distribution of surface glycoproteins on influenza A virus determined by electron cryotomography

**DOI:** 10.1016/j.vaccine.2012.09.082

**Published:** 2012-12-07

**Authors:** Sebastian Wasilewski, Lesley J. Calder, Tim Grant, Peter B. Rosenthal

**Affiliations:** Division of Physical Biochemistry, MRC National Institute for Medical Research, The Ridgeway, Mill Hill, London, NW7 1AA, United Kingdom

**Keywords:** HA, hemagglutinin, NA, neuraminidase, M1, matrix protein, RNP, ribonucleoprotein particle, Electron cryomicroscopy, Influenza virus, Hemagglutinin, Neuramindiase, RNP, Neutralizing antibody

## Abstract

We use electron cryotomography to reconstruct virions of two influenza A H3N2 virus strains. The maps reveal the structure of the viral envelope containing hemagglutinin (HA) and neuraminidase (NA) glycoproteins and the virus interior containing a matrix layer and an assembly of ribonucleoprotein particles (RNPs) that package the genome. We build a structural model for the viral surface by locating copies of the X-ray structure of the HA ectodomain into density peaks on the virus surface. We calculate inter-glycoprotein distances and the fractional volume occupied by glycoproteins. The models suggest that for typical HA densities on virus, Fabs can bind to epitopes on the HA stem domain. The models also show how membrane curvature may influence the number of glycoproteins that can simultaneously interact with a target surface of receptors.

## Highlights

► Cryotomography of influenza A virus reveals a polarized structure. ► 3D models for the virus envelope are built using glycoprotein X-ray structures. ► Inter-glycoprotein distances and fractional surface volumes are calculated. ► Accessibility of the HA stem domain to neutralizing antibodies is assessed. ► Viral membrane curvature may constrain interaction with a surface of receptors.

## 1 Introduction

The structural understanding of the influenza A virus life cycle and disease has been informed by high resolution crystal structures of the hemagglutinin [1], which binds cell surface receptors containing sialic acid and mediates membrane fusion during entry, and the neuramindase glycoprotein [2,3], the receptor-destroying enzyme that hydrolyzes sialic acid from receptors. These crystallographic studies have been complemented by ultrastructural studies of virions using negative stain electron microscopy and more recently by cryomicroscopy of frozen-hydrated specimens that preserves native structure. Electron cryotomography provides a further advance in our understanding of influenza virus ultrastructure by reconstructing three-dimensional maps of the frozen-hydrated specimen [4,5]. The resulting reconstructions are at considerably lower resolution than X-ray crystal structures because of radiation damage due to the requirement of recording many images of the same specimen. Furthermore, limited tilt angles cause blurring in one direction. Therefore interpretation and modeling must take into account the anisotropic resolution of the maps. Nevertheless, the interpretation of three-dimensional maps with X-ray structures creates a molecular model of virus architecture.

Here we describe three-dimensional maps of A/Aichi/68 X-31 and A/Udorn/72 virions determined by electron cryotomography. The latter strain maintains a filamentous phenotype in the laboratory and displays a structural regularity that may be exploited for structural study [4,6]. We build a model for the virus surface glycoproteins by placing X-ray models for the HA ectodomain at glycoprotein positions in the map. The models define structural parameters for the virus that have important consequences for understanding viral infection and the host immune response.

## 2 Materials and methods

Growth, purification, and cryotomography of A/Udorn/72 and A/Aichi/68 X-31 virus were done as previously described [4]. Structural models of the virus envelope were constructed by selecting cylindrical regions of virions and placing the X-ray models (pdb id 1HGE) into spike density perpendicular to the surface. Intermolecular distances were calculated between the centers-of-mass of the HA models (78 Å from membrane). For studies of FI6 Fab binding [7], the model (pdb id 3ZTJ) with different numbers of Fabs bound was examined for overlap with other HA models. To measure the relative distance of receptor binding sites, the O2 position of the sialic acid in the receptor-binding site was determined for all HA coordinates built on the virus surface.

## 3 Results

### 3.1 Cryotomography of influenza A virus

**Fig. 1:**
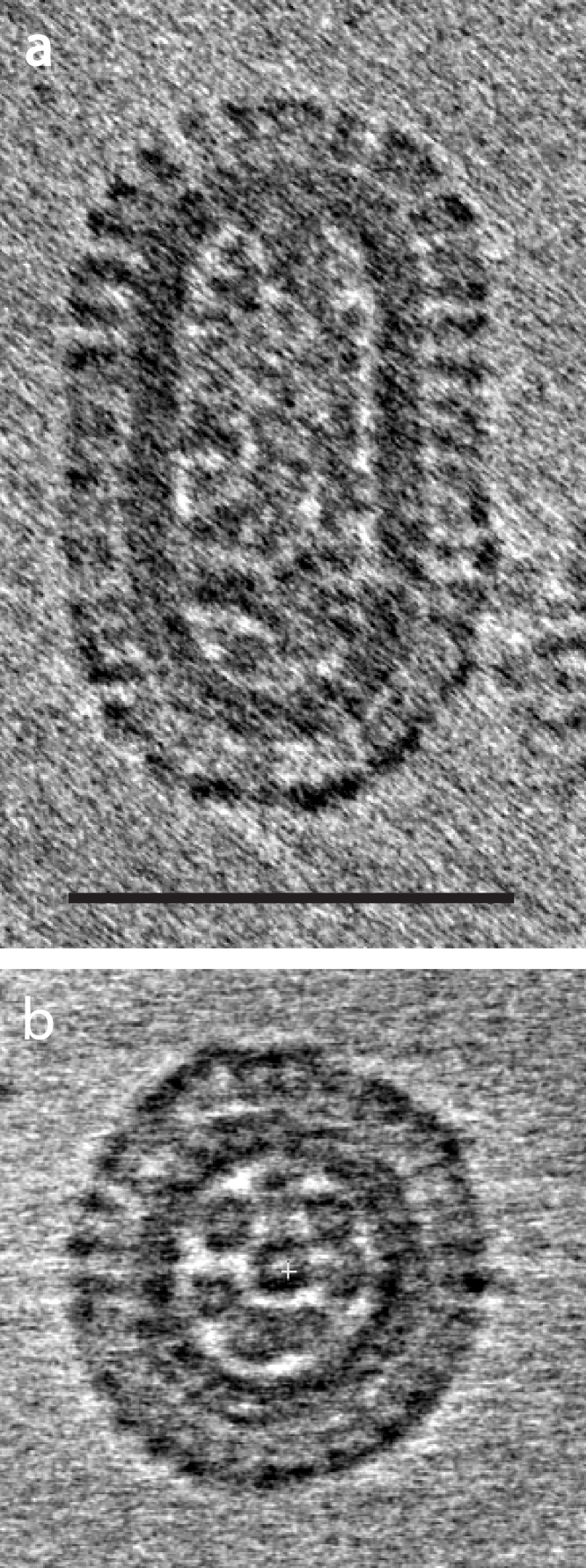
Tomogram sections of A/Udorn/72 virions. (a) A projection of a 130 Å thick slab through a capsule-shaped virion showing RNPs in interior, matrix layer adjacent to the virus membrane, and envelope glycoproteins. HA covers most of the virion, but NA clusters are visible at the bottom end. (b) Same-sized section of a different virion down the long axis of the particle showing RNPs in a 7 + 1 arrangement. Scale bar is 100 nm.

Cryotomography was used to study the three-dimensional structure of frozen-hydrated influenza virions (H3N2). Udorn virions typically show a capsular (cylindrical with hemispherical caps at the ends) or filamentous morphology. Fig. 1a shows a tomogram slice of a capsule-shaped Udorn virion with its long axis lying in the plane of the ice film. RNPs run the length of the virion inside the lipid bilayer, which is lined on the inside with a layer of the M1 protein, and on the outside by glycoprotein spikes. Less frequently the small capsular particles are oriented with their long axis perpendicular to the ice film (Fig. 1b). This shows the envelope glycoproteins and a layer formed by the M1 surrounding eight RNPs in a 7 + 1 arrangement previously identified in plastic sections of budding virus [8,9] which likely correspond to the eight genomic segments. In more elongated Udorn virions these are observed to be at one end [4].

We identify glycoproteins as strong densities with distinct features at the highest radius of the particles beyond the membrane. The HA glycoproteins are 13 nm long spikes with a density profile similar to the X-ray crystal structure of the trimeric ectodomain. The NA is 14 nm long and has density concentrated in the tetrameric head domain similar in size and shape to the crystal structure, located at the membrane distal end of a thin stalk. Clusters of NAs [4,5,10] are often seen at one end of the virion producing pronounced arcs of density 14 nm from the membrane (Fig. 1a). In elongated particles, it is clear that the clusters are at the end opposite to where the RNP assembly is observed [4]. The glycoproteins may interact with the matrix layer, but molecular features cannot be distinguished at the resolution of the tomograms. In summary, Udorn particles are cylindrical with RNPs near one hemi-spherical cap and clusters of NA are commonly observed on the surface of the hemi-spherical cap opposite the RNPs.

### 3.2 Glycoprotein distribution on the virus surface

**Fig. 2:**
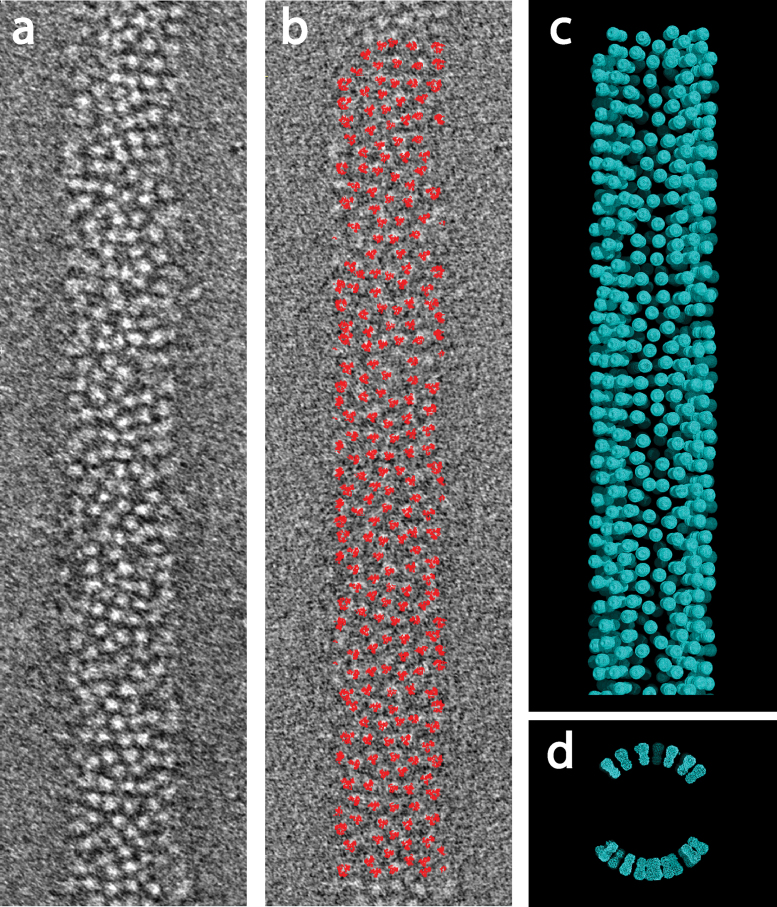
Glycoprotein positions on the virus surface. (a) Tomogram section of Udorn virions showing glycoproteins. (b) Glycoprotein positions identified by red model positions. (c) Surface mesh for HAs placed at density peaks. (d) End-on view of the model in (c) shows that glycoprotein models have only been built on the upper and lower cylindrical surfaces of the virion.

We build a structural model for the virus envelope by placing the X-ray model for the HA ectodomain at peak density positions on the virus membrane. Because of the anisotropic resolution of the tomograms due to the missing data wedge, the images of the virus surface are blurred along the direction of the membrane at the sides of the particles, which cannot be tilted toward the electron beam. For this reason, we only build models for the glycoproteins on the top and bottom cylindrical surfaces of the virus and restrict our analysis to these surfaces. These positions are indicated for a Udorn virion in Fig. 2. Because we cannot always distinguish the orientation of the trimeric spikes about their axis, we describe the glycoprotein positions by an envelope calculated from cylindrically averaged density for the X-ray structure. While some of the density peaks that we model as HAs could instead be NAs, which are present in much smaller numbers than the HAs, this will not affect the average properties that we describe for the viral envelope or the conclusions below. We have not modeled the NA clusters at the hemispherical poles of the virion.

**Fig. 3:**
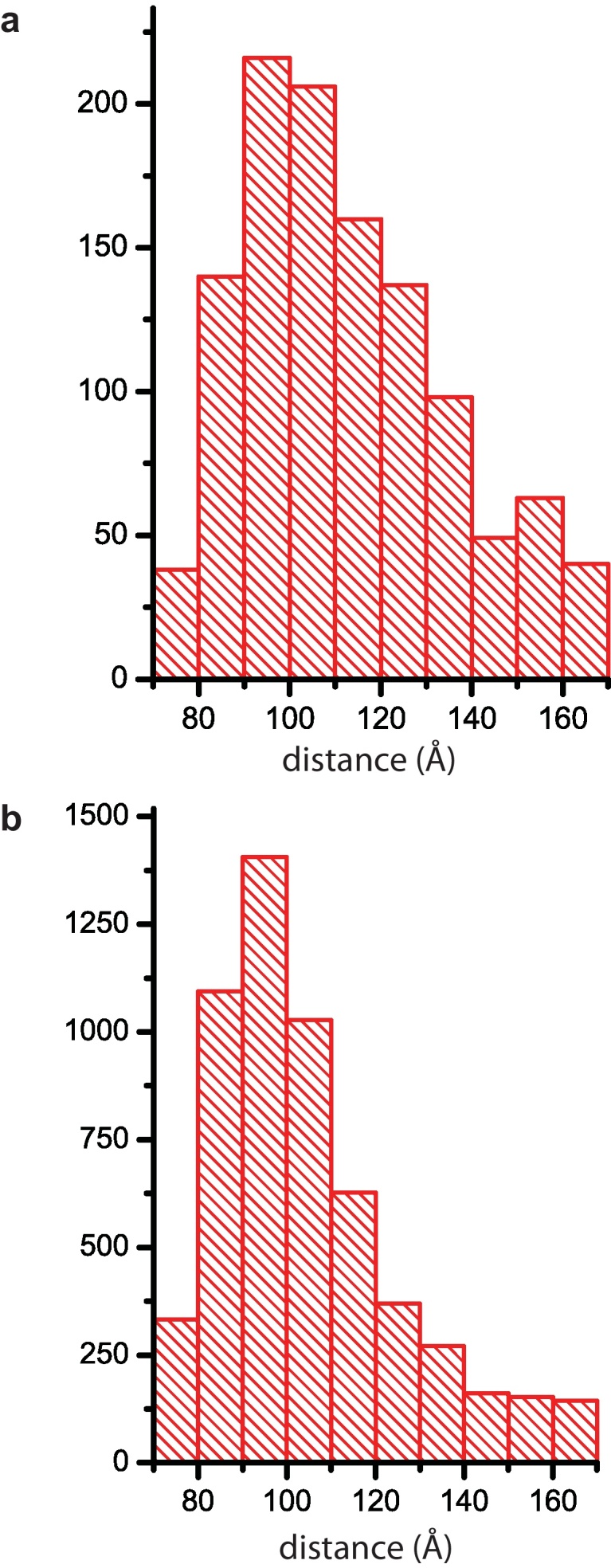
Histogram of inter-glycoprotein spacing. (a) X-31 histogram shows 1147 inter-spike distances on 3 virions. The closest 5 nearest neighbor distances are included. (b) Udorn histogram shows 5583 inter-spike distances on 3 virions, again showing the closest 5 nearest neighbor distances.

We measure the distance between each glycoprotein position and its five nearest neighbors on both X-31 and Udorn virions and plot these as separate histograms in Fig. 3. The histograms peak at 91 Å in each case. The X-31 mean spacing (112 Å ± 23 Å) is similar to that reported in an earlier cryotomography study [5]. The Udorn spacing is similar (104 Å ± 20 Å) but has a narrower distribution, consistent with its more dense and ordered appearance. The difference of the mean from the peak value is due to the long tails of the distribution for large distances that are the effect of small gaps in the glycoprotein positions. The HA glycoproteins are 70 Å at their widest and are therefore well-separated on average and not in contact at their ectodomains. Based on our models of the HAs, we calculate the fractional volume occupied by the glycoproteins on the surface, defined here as a layer beyond the membrane one HA molecule thick. The fractional volume values for the three X-31 virions reported in Fig. 3 are 13.5%, 15.0%, and 15.5% and for the three Udorn virions, 15.2%, 16.8%, and 19.2%. The fraction of the membrane surface area that the HA covers in projection is roughly twice the volume fraction value, and reflects the fact that the HA deviates from a cylinder in shape so that the head domain hides volume close to the membrane.

**Fig. 4:**
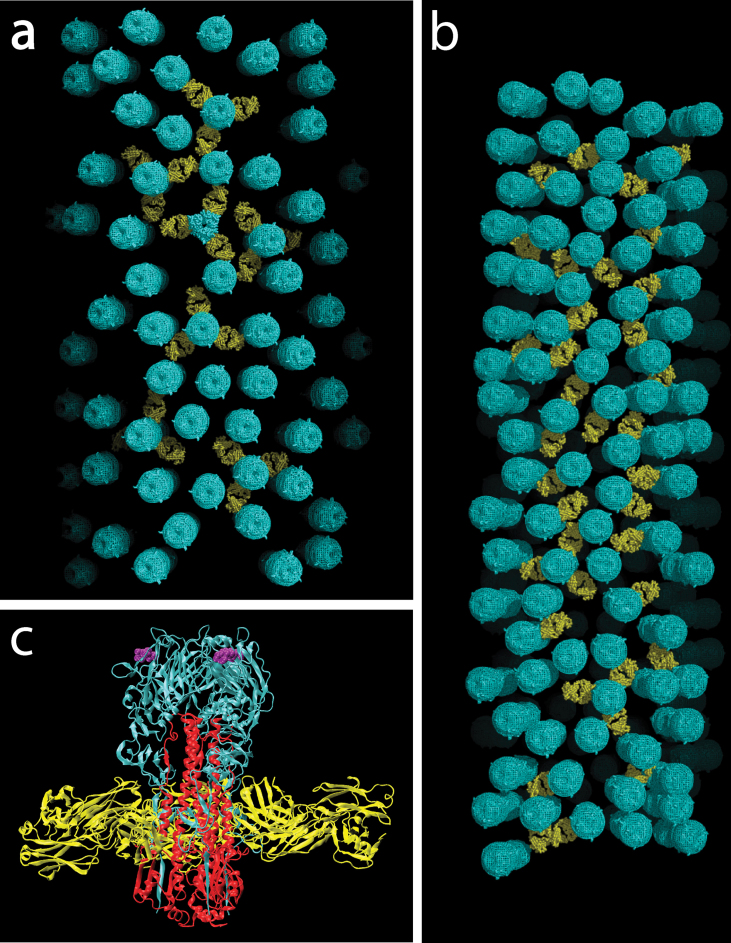
(a) Glycoprotein positions for an X-31 virion with 13% fractional surface volume. Glycoproteins are represented as cylindrically averaged molecules (cyan) to reflect orientational uncertainty. Several models show 3 FI6 Fabs (yellow) bound to the HA stem. (b) Glycoprotein positions for a Udorn virion with 15% fractional volume. (c) X-ray model of HA trimer with FI6 Fabs bound to stem domain [7].

Fig. 4a shows a model for the glycoprotein positions on one surface of an X-31 virion with a fractional volume of 13%. The surface is surprisingly open in contrast to the impression from viewing the virus in projection images. Because the HA is recognized by neutralizing antibodies, we considered which parts of the protein are accessible to antibodies in the context of the virus surface. While the sequence variable head domain is likely to be exposed, one consequence of the open packing is that epitopes near the membrane are accessible. Fig. 4c shows the previously described crystal structure [7] of the HA in complex with an Fab from the broadly neutralizing antibody FI6 that recognizes an epitope in the stem domain. In Fig. 4a, several HA positions are shown where there is enough room for 3 Fabs to bind a single HA without clashing into another HA position. Fig. 4b shows a Udorn surface of slightly higher fractional volume (15%). Several positions are also shown where there is enough room for an HA to bind a single Fab, and typically each glycoprotein can be oriented to bind at least one Fab. Though we have assessed the locations where Fabs can bind using a rigid Fab model, when the known flexibility of the Fab is considered, there are likely to be even fewer constraints on binding the stem region.

### 3.3 Virion curvature and glycoprotein orientation

**Fig. 5:**
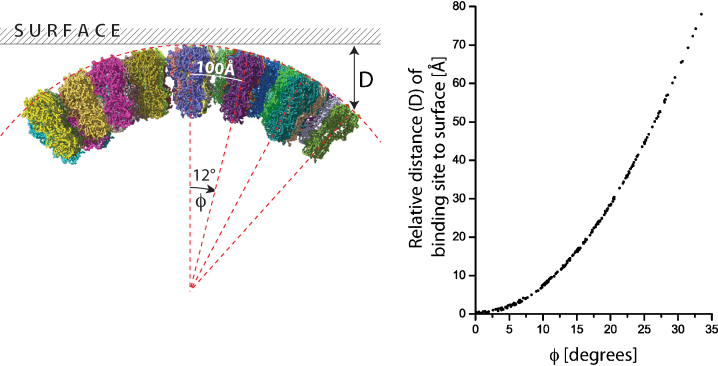
Distance (*D*) of HA receptor binding sites from a planar surface of receptors as a function of the HA position along the curved cylindrical virus surface described by the angle *ϕ*⋅ *ϕ* is 0̊ at closest approach to the surface. Each point of the graph represents D for one of the receptor binding sites (three per HA) in the model of the virion glycoproteins. The ∼100 Å separation between glycoproteins on the virus corresponds to a *ϕ* increment of 12̊ as marked in the angle diagram.

A striking feature of the virus particles is the curvature of the membrane. For capsule or filament-shaped viruses of the most typical dimension in our preparations, the virus has a small radius of curvature perpendicular to the long axis of the capsule (Fig. 5). One consequence of this curvature would be a geometric constraint on the fraction of the virus surface that could engage with receptors on a target surface. The receptor binding site is located near the top of the HA as shown by the purple ligand in Fig. 4c. We calculate the relative distance of the receptor binding sites (Fig. 5) from a planar surface as an increasing angle *ϕ* about the cylindrical axis of the particle from the point of closest approach. Note that for the typical spacings described above, the ∼100 Å distance between spikes corresponds to a relative angle of 12̊. Assuming at least one HA can engage receptors on a surface, then the binding sites of the next closest HA are on average 12 Å further away from the surface. For a spherical-shaped particle, different directions of curvature are identical. In the case of capsular or filamentous particles, HAs along the axis maintain the same distance and could simultaneously engage receptors.

## 4 Discussion

Cryotomography of the influenza virus X-31 [4,5] and Udorn [4] has revealed the three-dimensional structure of the virus envelope containing glycoproteins, the virus interior containing an assembly of RNPs packaging the genome, and a dense matrix layer inside the viral membrane. Though influenza virus is pleomorphic, a large fraction of particles are ellipsoidal with hemispherical ends. Compared to X-31, the Udorn particles have more uniform diameters, and have a narrower and cylindrical shape. These have been attributed to strong stabilizing interactions in the matrix layer [4,11] that confer a filamentous morphology. Image analysis has shown that for the most-ordered Udorn particles the matrix layer is a helical organization of the M1 protein. When the virus is incubated at low pH, cryomicroscopy shows that a loss of filamentous morphology is associated with the matrix layer being driven-off the membrane and forming a dense multi-layered coil structure.

The images in Fig. 1 capture the main features of influenza virus structure and assembly, showing a polarized structure with RNPs aligned along the cylindrical axis of the particles, and NA clusters at one end of the virion. In elongated particles the NA clusters are observed at the opposite end from where RNPs are observed. Microscopy of virus budding from infected cells shows the RNP assembly is at the apical end [9] and therefore NA clusters are near the point of pinching-off. Once budding is initiated, HAs likely interact with the polymerizing matrix layer to determine the elongated morphology of the virions. NA incorporation may define the end of the budding process by disrupting HA-matrix polymerization. The M2 ion channel protein is also localized to this end of the virus during budding [12,13], but is too small to resolve by cryotomography. These observations are consistent with membrane glycoproteins all playing a role in determining virus morphology [14].

Earlier studies of the surface glycoprotein density have relied upon bulk scattering methods such as neutron diffraction [15]. While glycoprotein density has been estimated from glycoproteins at the edge of single projection images [16,17], tomography is more accurate because it avoids problems of molecular overlap by calculating the three-dimensional structure [4,5]. We build structural models for the arrangement of the surface glycoproteins that assign the position and orientation of the HA X-ray structure but not a specific rotation about the three-fold axis. The structural models show that the glycoproteins are not close-packed. The strong crystalline order of the Udorn matrix layer does not appear to extend to the glycoproteins. However, the glycoprotein distribution in Udorn is more ordered than X-31 which points toward translational restriction of the HA and supports the idea of interactions with the matrix layer. Higher resolution analysis by tomography or biophysical measurement will be required to see whether there is any rotational ordering to the glycoproteins. Our model for the influenza glycoprotein distribution defines several structural parameters that may be important for understanding the virus life cycle as well as preventing infections with drugs and vaccines.

The structural models of the envelope glycoprotein on the virus surface suggest geometric constraints on receptor binding determined by the glycoprotein spacing and radius of curvature of the virus membrane. In vitro experiments indicate a weak millimolar binding constant of the HA glycoprotein for sialic acid receptors. Furthermore, influenza host specificity is dependent on very small affinity differences for sialic acid receptors with different glycosidic linkages [18,19]. Infection therefore depends on multivalent binding. The number of HAs that can simultaneously participate in binding will be a key determinant in virus entry. The curvature of the virus surface and spacing of glycoproteins determines the number of adjacent glycoproteins that can simultaneously engage receptors on a planar surface such as those used in in vitro binding studies. The flexibility, length, and density of lipids or proteins bearing sialic acid receptors on cells will influence the number of HAs engaged with receptors as will the rigidity and contour of the host membrane and its ability to wrap around the curved surface of influenza virus.

The three-dimensional structural models of the glycoprotein on the surface of influenza virions describe important structural parameters that govern antibody recognition of the HA including the density and accessibility of epitopes. The average glycoprotein spacing observed (∼100 Å) is short enough for bivalent IgGs, which possess flexibly linked antigen binding sites that can extend 150 Å apart, to cross-link adjacent HAs [20,21]. The off-rate for IgG binding decreases due to avidity, making viral escape from neutralizing antibodies through mutation more difficult [22]. Most neutralizing antibodies recognize sites on the sequence variable globular head domain of HA that are likely to be accessible on the virus surface and block cell attachment by preventing receptor binding [23]. There has been recent interest in broadly neutralizing antibodies that bind to conserved features on the HA [7,24,25]. The plasma cell-derived antibody FI6 recognizing the less sequence variable stem of the HA has been shown to be a broadly neutralizing antibody of all known influenza strains (group 1 and group 2) [7]. Our structural models for the H3N2 virus surface suggest that there is enough space for the Fab to bind the HA. The glycoprotein spacing reported for H1N1 viruses [16] suggests that this observation can likely be extended to both group 1 and group 2 viruses. Therefore, these Fabs can bind the HA on the virus surface in addition to HA expressed on the surface of infected cells. Despite their flexibility, the efficiency of binding by IgGs may be further reduced by the shielding of the stem regions by the HA head domain. An understanding of the three-dimensional structural arrangement of the glycoproteins may therefore be applied in vaccine and drug design, including to antibodies that recognize and block membrane fusion rather than receptor binding.

## 5 Conclusions

The three-dimensional maps of influenza virus determined by electron cryotomography show the packaging of the genomic segments in the virus interior and the envelope structure including a dense matrix layer inside the bilayer and glycoproteins outside. We have used X-ray structures of the HA to build three-dimensional models for the surface glycoprotein distribution that show large scale structural features that are likely to be important for understanding of the virus life-cycle. Electron cryotomography can also be applied to visualize neutralizing antibodies in complex with virus and viruses interacting with target membranes.
